# Mass spectrometry database of lacrimal gland adenoid cystic carcinoma and normal lacrimal gland tissue identifies extracellular matrix remodeling in these tumors

**DOI:** 10.1016/j.dib.2023.109330

**Published:** 2023-06-19

**Authors:** Acadia H.M. Moeyersoms, Vasileios Stathias, Ravi Doddapaneni, David T. Tse, Daniel Pelaez

**Affiliations:** a Dr. Nasser Ibrahim Al-Rashid Orbital Vision Research Center, Bascom Palmer Eye Institute, University of Miami Miller School of Medicine, Miami, FL, USA; b Sylvester Comprehensive Cancer Center, University of Miami, Miller School of Medicine, Miami, FL, USA; c Department of Pharmacology, University of Miami, Miller School of Medicine, Miami, FL, USA; d McColl-Lockwood Laboratory for Muscular Dystrophy Research, Atrium Health Musculoskeletal Institute, Wake Forest School of Medicine, Carolinas Medical Center, Charlotte, NC, USA; e Department of Biomedical Engineering, University of Miami College of Engineering, University of Miami, Miami, FL, USA

**Keywords:** Cancer research, Ocular oncology, Proteomics, Pathway analysis

## Abstract

Adenoid cystic carcinoma of the lacrimal gland (LGACC) is a slow-growing but aggressive orbital malignancy. Due to the rarity of LGACC, it is poorly understood, which makes diagnosing, treating, and monitoring disease progression difficult. The aim is to understand the molecular drivers of LGACC further to identify potential targets for treating this cancer. Mass spectrometry was performed on LGACC and normal lacrimal gland samples to examine the differentially expressed proteins to understand this cancer's proteomic characteristics. Downstream gene ontology and pathway analysis revealed the extracellular matrix is the most upregulated process in LGACC. This data serves as a resource for further understanding LGACC and identifying potential treatment targets. This dataset is publicly available.

**Specifications Table**

**Table:** 

Subject	Cancer Research
Specific subject area	Ocular oncology, proteomics
Type of data	Table Graph Figure
How the data were acquired	Mass spectrometry, MaxQuant software v1.6.2.3, limma package 3.54.0, DAVID Functional Analysis Tool
Data format	Raw Analyzed Filtered
Description of data collection	LGACC and normal lacrimal gland samples were flash frozen. Mass spectrometry was conducted using label-free analysis with 4h LC/MS/MS. Data were processed with MaxQuant software v1.6.2.3, and further analyzed with limma 3.54.0 and DAVID Functional Analysis Tool.
Data source location	• Institution: Bascom Palmer Eye Institute, University of Miami Miller School of Medicine • City/Town/Region: Miami, FL • Country: USA
Data accessibility	Repository name: Mendeley Data Data identification number: 10.17632/ppx5hyfchh.1 Direct URL to data: *https://data.mendeley.com/datasets/ppx5hyfchh* Repository name: GitHub Data identification number: 10.5281/zenodo.7915576 Direct URL to data: *https://zenodo.org/badge/latestdoi/638497288*
*Related research article*	None

## Value of the Data

• This data provides differential protein expression between LGACC and normal lacrimal glands. It provides a deeper understanding of this cancer's molecular composition and processes.
• This data is beneficial to researchers studying LGACC and those interested in orbital malignancies.
• This data provides a dataset of potential protein targets for advancing the clinical management of LGACC.

## 1 Objective

We conducted mass spectrometry analysis of adenoid cystic carcinoma of the lacrimal gland (LGACC) and normal lacrimal gland tissue to identify differential proteomic expression signatures between the cancerous tissue and healthy tissue. There is little known about the molecular signatures of this rare cancer, and we wanted to add to the field by analyzing the protein characteristics.

## 2 Data Description

**Fig. 1:**
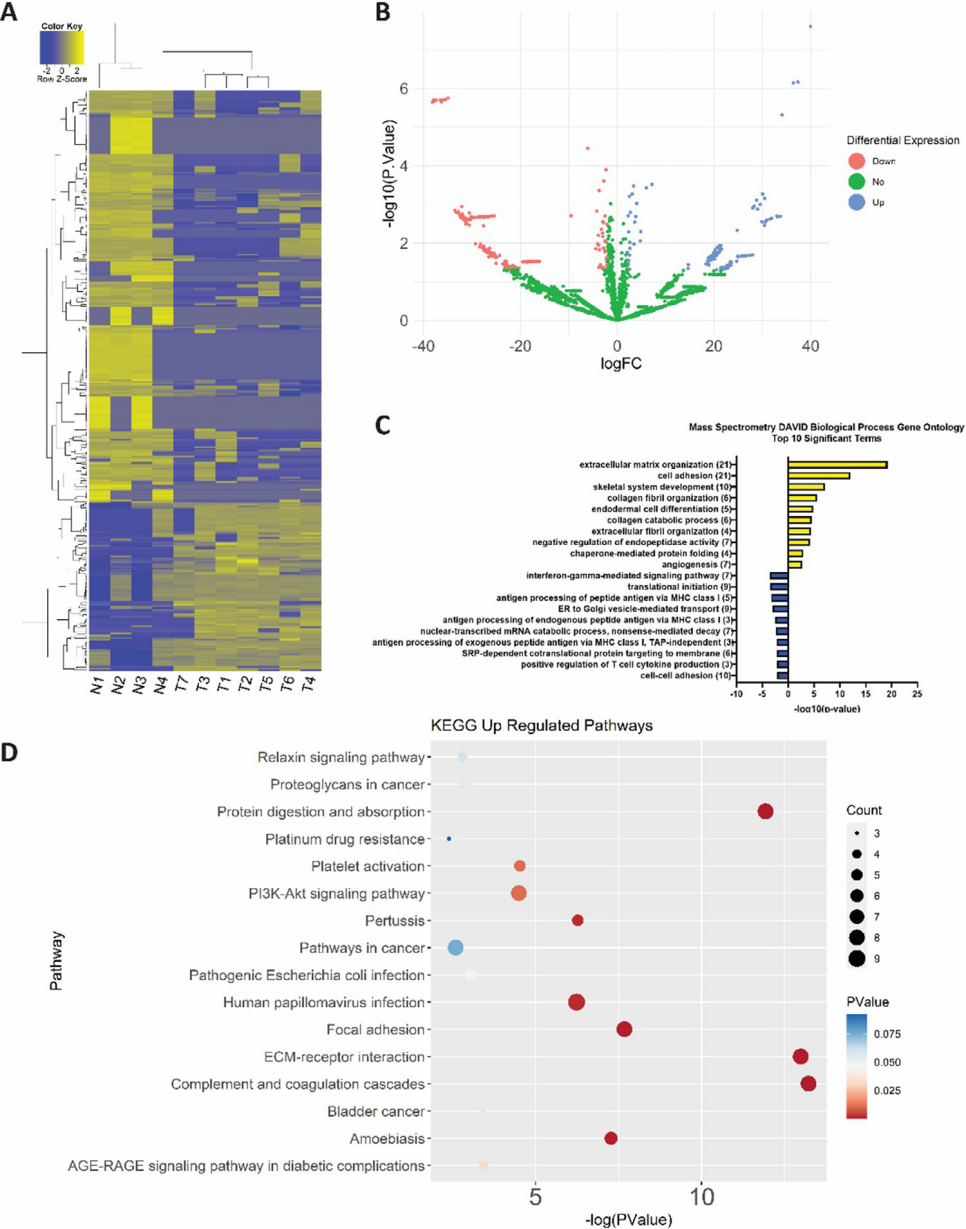
Mass spectrometry analysis of LGACC verses normal lacrimal gland. (A) Heatmap of all proteins in mass spectrometry analysis of normal samples (n = 4) vs. LGACC samples (n = 7). (B) Volcano plot of logFC by -log10(pValue) showing differentially expressed genes with logFC cut off of 2 and -2 and raw pValue > 0.05. Red and blue dots represent downregulated and upregulated genes, respectively. (C) Biological process gene ontology analysis of up and down-regulated genes. (D) KEGG pathway analysis of upregulated proteins. (For interpretation of the references to color in this figure legend, the reader is referred to the web version of this article.)

**Fig. 2:**
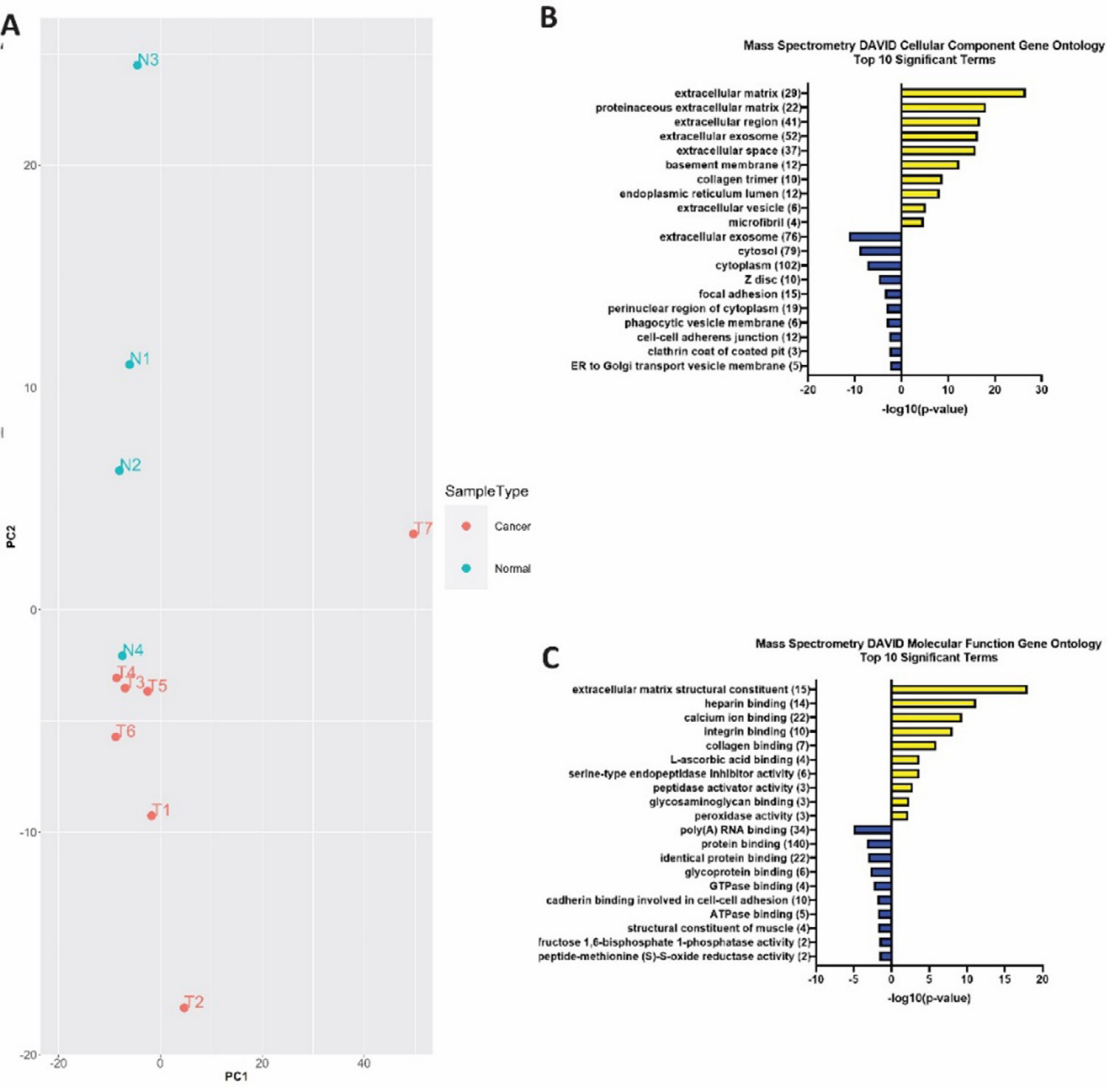
LGACC and normal sample comparisons via Mass spectrometry downstream analysis. (A) PCA plot of 11 Mass spectrometry samples. Red and teal represent cancer and normal samples, respectively. (B) Cellular component gene ontology analysis of up and down-regulated genes. (C) Molecular function gene ontology analysis of up and down-regulated genes. (For interpretation of the references to color in this figure legend, the reader is referred to the web version of this article.)

The findings of differential protein expression between the normal lacrimal gland (n = 4) and LGACC (n = 7) are presented. Mass spectrometry was performed on primary tumor samples to identify the proteomic differences between cancerous and normal tissue. The raw values that were processed in the MaxQuant software are in the data repository Mendeley Data (Title: Mass Spectrometry of lacrimal gland adenoid cystic carcinoma and normal lacrimal gland) in the file Raw_MassSpec_Data. The R Studio package limma was used to determine the differentially expressed proteins between normal and cancerous samples. The output of the limma analysis is in the data repository file MassSpect_limma_output including the raw values and the statistical analysis outputs. Three hundred eighty-three (383) differentially expressed proteins between LGACC and normal lacrimal glands were identified, 111 upregulated genes, and 272 downregulated genes. The differentially expressed genes (n = 383) including the raw values and the p-values can be found in the data repository file MassSpec_Analyzed_DEGs. Fig. 1A shows the heatmap of the log2 transformed raw data of all samples. Fig. 1B depicts a volcano plot representing all the proteins that are downregulated (red), upregulated (blue), and not differentially expressed (green) in LGACC compared to normal lacrimal gland, with the -log2 of the raw p-value on the y-axis and the log of the fold change on the x-axis. Fig. 2A is a principal component analysis (PCA) plot showing the clustering of the samples based on PCA analysis. Downstream gene ontology analysis using DAVID functional tool analysis was conducted to determine the significant ontologies in LGACC. The up and down regulated ontologies for LGACC compared to normal lacrimal gland are represented in: Fig. 1C for biological processes, Fig. 2B for cellular components, and Fig. 2C for molecular functions. Fig. 1D presents the up-regulated KEGG pathways in LGACC. Mass spectrometry analysis of LGACC provides a data resource for future work to be done in understanding the molecular makeup of this cancer and identifying potentially actionable targets for novel treatments.

## 3 Experimental Design, Materials and Methods

### 3.1 Sample preparation

Normal lacrimal gland and LGACC tissue samples were extracted during surgery. Flash-frozen tissue samples were sent to MS Bioworks (Ann Arbor, MI) for sample preparation and mass spectrometry using label-free analysis with 4h LC/MS/MS per sample.

### 3.2 Protein identification and differential gene expression analysis

Samples were analyzed with a Waters NanoAcquity HPLC system with ThermoFisher Fusion Lumos. Data were processed with MaxQuant software v1.6.2.3. Uniprot names were converted to gene names using the Uniprot website (4,024 genes). Duplicate gene names were removed (3,995) by filtering based on the primary gene name. PCA, heatmap, and volcano plots were made in R Studio. For PCA analysis, a value of 1 was added to all raw values, and the log2 was taken of all values. Further downstream analysis was processed with the R package limma (Version 3.54.0) [1]. The limma analysis performed was used to calculate the *p*-value, adjusted *p*-value, and log fold change [2]. Proteins that had a p-value of <0.05 and a logFC >2 or less than <2 were considered significant.

### 3.3 Gene ontology and pathway analysis

The differentially expressed genes found in the mass spectrometry data (n = 383) were analyzed for downstream gene ontology and pathway analysis using DAVID Functional Analysis Tool [3,4]. Ontology figures were made using the -log10 of the p-value for each ontology.

## Ethics Statement

The University of Miami Institutional Review Board approved this study (IRB 20090524). The study was conducted in a Health Insurance Portability and Accountability Act of 1996-compliant manner.

## CRediT authorship contribution statement

**Acadia H.M. Moeyersoms:** Conceptualization, Data curation, Formal analysis, Investigation, Methodology, Software, Validation, Visualization, Writing – original draft, Writing – review & editing. **Vasileios Stathias:** Data curation, Formal analysis, Methodology, Software, Writing – review & editing. **Ravi Doddapaneni:** Data curation, Investigation, Writing – review & editing. **David T. Tse:** Funding acquisition, Resources, Writing – review & editing. **Daniel Pelaez:** Conceptualization, Funding acquisition, Investigation, Project administration, Resources, Supervision, Writing – review & editing.

## Declaration of Competing Interest

The authors declare that they have no known competing financial interests or personal relationships that could have appeared to influence the work reported in this paper.

## Data Availability

Mass Spectrometry of lacrimal gland adenoid cystic carcinoma and normal lacrimal gland (Original data) (Mendeley Data).
